# Ti/Ni co-doped perovskite cathode with excellent catalytic activity and CO_2_ chemisorption ability *via* nanocatalysts exsolution for solid oxide electrolysis cell

**DOI:** 10.3389/fchem.2022.1027713

**Published:** 2022-10-10

**Authors:** Shuying Zhen, Lihong Zhang, Chunming Xu, Ding Zhang, Qun Yi, Wang Sun, Kening Sun

**Affiliations:** ^1^ State Key Laboratory for Advanced Metals and Materials, University of Science and Technology Beijing, Beijing, China; ^2^ Beijing Key Laboratory for Chemical Power Source and Green Catalysis, Beijing Institute of Technology, Beijing, China; ^3^ School of Chemical Engineering and Pharmacy, Wuhan Institute of Technology, Wuhan, China

**Keywords:** solid oxide electrolysis cells (SOECs), double perovskite oxide, cathode, *in situ* exsolution, CO_2_ reduction reaction

## Abstract

Carbon dioxide (CO_2_) gas is the main cause of global warming and has a significant effect on both climate change and human health. In this study, Ni/Ti co-doped Sr_1.95_Fe_1.2_Ni_0.1_Ti_0.2_Mo_0.5_O_6-δ_ (SFNTM) double perovskite oxides were prepared and used as solid oxide electrolysis cell (SOEC) cathode materials for effective CO_2_ reduction. Ti-doping enhances the structural stability of the cathode material and increases the oxygen vacancy concentration. After treatment in 10% H_2_/Ar at 800°C, Ni nanoparticles were exsolved *in situ* on the SFNTM surface (Ni@SFNTM), thereby improving its chemisorption and activation capacity for CO_2_. Modified by the Ti-doping and the *in situ* exsolved Ni nanoparticles, the single cell with Ni@SFNMT cathode exhibits improved catalytic activity for CO_2_ reduction, exhibiting a current density of 2.54 A cm^−2^ at 1.8 V and 800°C. Furthermore, the single cell shows excellent stability after 100 h at 1.4 V, indicating that Ni/Ti co-doping is an effective strategy for designing novel cathode material with high electrochemical performance for SOEC.

## Introduction

Carbon dioxide (CO_2_), a greenhouse gas and the main cause of global warming, has a significant impact on both climate change and human health. (Armaroli and Balzani, 2011; Broecker, 2018; Hoegh-Guldberg et al., 2019). The capture and utilization of CO_2_ is a promising approach to effectively reduce CO_2_ concentration and emissions (Fu et al., 2019; Karanikolos et al., 2021). It is known that CO can be further converted to low-carbon fuels and high value added chemicals through subsequent Fischer-Tropsch reactions (Zhang et al., 2002; Quadrelli et al., 2011). Therefore, converting CO_2_ to CO is a feasible and important method for CO_2_ utilization. Solid oxide electrolysis cells (SOEC) are high-efficiency electrochemical reactors that can convert CO_2_ into CO using distributed renewable energy, with an efficiency close to 100 % (Singh et al., 2015; Li et al., 2021). Normally, the CO_2_ reduction reaction (CO_2_-RR) occurs at the cathode of the SOEC, where CO_2_ is electrochemically converted to CO and oxygen ions under an applied potential. The generated oxygen ions are transferred across the solid electrolyte to the anode and converted to O_2_. The cathode is the core component in the SOEC, and dominates the electrochemical CO_2_-RR process and the performance of the SOEC.

Presently, Ni-YSZ is the most widely used cathode in SOEC for CO_2_-RR, owing to its good electrocatalytic activity and cost effectiveness (Song et al., 2018; Xu et al., 2019). However, certain inevitable issues hinder its further application, including Ni oxidation during the CO_2_/CO redox reaction process in the absence of a safe gas (H_2_/CO), carbon deposition at high CO concentrations, and Ni particle growth during long-term operation (Dong et al., 2017; Sciazko et al., 2021; Yang et al., 2022). Materials comprising perovskite oxide with mixed ionic-electronic conductivity, such as Sr_2_Fe_1.5_Mo_0.5_O_6-δ_ (SFM), show promise for SOEC, and exhibit excellent redox stability, coke resistance, and long-term stability (Li et al., 2017a; Zhang et al., 2021). Nevertheless, compared with the Ni-YSZ cathode material, SFM oxide exhibits weak chemical CO_2_ adsorption and inadequate catalytic activity for CO_2_-RR, which limits the performance of CO_2_ electrolysis. Recently, the *in situ* exsolution of transition metal nanoparticles on the surface of perovskite oxide substrates has attracted extensive research attention and is regarded as a promising strategy to enhance the electrocatalytic activity of perovskite oxides. (Wang et al., 2019; Xu et al., 2021)

Briefly, the exsolved metal nanocatalysts, uniformly dispersed on the surface, can effectively strengthen the adsorption capacity of CO_2_ and improve the electrolysis activity for CO_2_-RR. Abundant oxygen vacancies were created during the reduction process, thereby supplying more active reaction regions for the chemisorption and catalytic activation of CO_2_ molecules on the electrode surface (Liu S. B. et al., 2016; Zheng et al., 2017). Furthermore, the metal nanoparticles were firmly anchored on the surface of the electrode material, restricting their aggregation. The strong interaction of the metal-oxide interface also exhibits exceptional electrocatalytic activity for the CO_2_-RR. Chen et al. concluded that exsolved NiFe alloy nanoparticles in Sr_1.9_Fe_1.5_Mo_0.4_Ni_0.1_O_6-δ_ enhanced the chemisorption capacity and reaction kinetics of CO_2_ on the cathode surface (Wang et al., 2016). Bao et al. demonstrated that *in situ* exsolved FeNi_3_ nanoparticles on the Sr_2_Fe_1.35_Mo_0.45_Ni_0.2_O6_6-δ_ material strengthened CO_2_ adsorption and facilitated subsequent CO_2_-RR in SOEC (Lv et al., 2019). Luo et al. confirmed that *in situ* exsolved Fe-Ni nanospheres are evenly anchored on the perovskite oxide with oxygen defects, greatly enhancing the catalytic performance of the material as a highly stable and efficient catalyst (Liu S. et al., 2016). However, SFM-based double perovskite oxides tend to transform into Ruddlesden-Popper (RP)-type layer perovskite oxides during the *in situ* exsolution process in the reduction treatment, causing the initial perovskite structure to deform (Park et al., 2019; Park et al., 2020; Choi et al., 2021). The structural stability can be enhanced by employing stable valence state metallic elements, such as Ti^4+^, Zr^4+^, and Nb^5+^ in severe reducing atmospheres. For example, we introduced a stable valence Ti element to the B-site of SFM oxide, which notably improved its structural stability, produced abundant oxygen vacancies and increased the conductivity of the oxygen ions (Xu et al., 2022).

Herein, a Ti and Ni co-doped double perovskite oxide (Sr_1.95_Fe_1.2_Ni_0.1_Ti_0.2_Mo_0.5_O_6-δ_, SFNTM) was synthesized. After the reduction treatment, the oxide matrix maintained the original perovskite structure, while *in situ* exsolved Ni nanoparticles were uniformly anchored on its surface to form a metal-oxide heterostructure (Ni@SFNTM) as SOEC cathodes for CO_2_-RR at high temperatures. Subsequently, the phase structure, CO_2_ chemisorption and activation, and electrochemical properties of SFNTM and Ni@SFNTM samples were studied. The heterostructure formed by the *in situ* exsolved Ni nanoparticles and the SFNTM matrix can enhance the CO_2_ adsorption activity and expand the abundant CO_2_-RR active sites, thus further enhancing its performance.

## Experimental section

### Material preparation and cell fabrication

An Sr_1.95_Fe_1.2_Ni_0.1_Ti_0.2_Mo_0.5_O_6-δ_ (SFNTM) sample was prepared using a modified sol-gel combustion method. The calculated stoichiometric ratios of Ni(NO_3_)_2_·6H_2_O, Sr(NO_3_)_2_, Fe(NO_3_)_3_·9H_2_O, and (NH_4_)_6_Mo_7_O_24_·4H_2_O were added to deionized water and stirred at 80°C. C_16_H_36_O_4_Ti was dissolved in ethanol, and the content was added dropwise into the abovementioned solution. Subsequently, citric acid and glycine were added, and a gel was obtained *via* continuous mixing. A black precursor powder was obtained by heating the gel at 250^°^C followed by sintering at 1,100°C for 5 h to synthesize SFNMT oxide. The La_0.6_Sr_0.4_Co_0.2_Fe_0.8_O_3_ (LSCF) cathode, Ce_0.8_Sm_0.2_O_2_ (SDC), and La_0.8_Sr_0.2_Ga_0.8_Mg_0.2_O_3_ (LSGM) electrolyte materials were purchased from Fuel Cell Co. The Ni@SFNTM sample was prepared by reducing the SFNTM in 5 % H_2_/Ar at 800^°^C for 5 h.

An electrolyte-supported single cell (SFNTM/Ni@SFNTM|SDC|LSGM|LSCF) was used. Dense disk-shaped LSGM electrolyte flakes (∼300 μm) were formed by dry-pressing and calcined at 1,450^°^C for 6 h. The SDC slurry was formulated by mixing SDC powder and a binder comprising a mixture of ethyl cellulose and α-terpineol, which was screen-printed onto the two sides of the LSGM electrolyte pellet and subsequently calcined at 1,350^°^C for 3 h. The LSCF and SFNTM/SFTM ink were formulated by combining the LSCF and SFNTM/SFTM powders with a binder. The LSCF anode and SFNTM/SFTM cathode ink were symmetrically screen-printed onto both sides of the electrolyte pellet and calcined at 1,100^°^C for 2 h. Silver ink and silver wires were used to cover the electrode surface and the electrode was fired at 750^°^C for 1 h to act as the current collector.

### Characterization

The crystal structures of the as-synthesized and reduced SFNTM samples were investigated using X-ray diffraction (XRD, X'Pert Pro MPD diffractometer). The morphologies and microstructures of the reduced samples were observed using field-emission transmission electron microscopy (FETEM, JEM-2010F) and Scanning electron microscopy (SEM, FEI QUANTA-250). XPS (MULT1LAB 2000) was conducted to characterize the surface valence compositions of the different elements. Temperature-programmed desorption of CO_2_ (CO_2_-TPD) measurements were performed using a Micromeritics 2000 instrument with an Ar carrier gas, and the temperature was increased from 50°C to 1,000°C at a rate of 10°C min^−1^. The prepared materials were first treated under a He atmosphere at 300°C. The oxygen vacancy defects were detected by EPR (Bruker ELEXSYS E500). EPR tests were performed at 600 K with 10 mg sample and the sweep width is 1000G. The synthesized samples were tested by a four-probe direct current method. The Keithley 2,400 source meter was used to test on the dense bar shapes with a size of 2 mm × 4 mm×10 mm, which were prepared by dry pressing method and subsequently sintered at 1,200°C for 3 h, resulting in a density of over 95%. Electrical conductivity relaxation (ECR) measurement were performed under an abrupt switch of the atmosphere from 2:1 CO−CO_2_ to 1:1 CO−CO_2_. The variations in conductivity and test time were carried out until new equilibrium conditions are finally reached. Electrochemical impedance spectroscopy (EIS) of a single cell was performed using an AutoLab 302N at an open circuit voltage (OCV) by passing pure CO_2_ gas into the cathode at a flow rate of 50 ml min^−1^ while directly exposing the anode side to ambient gas. The corresponding AC impedance was analyzed using ZSimpWin software. The composition of the exhaust gas was evaluated using the online gas chromatography.

## Results and discussion

### Material structural characterization

**FIGURE 1:**
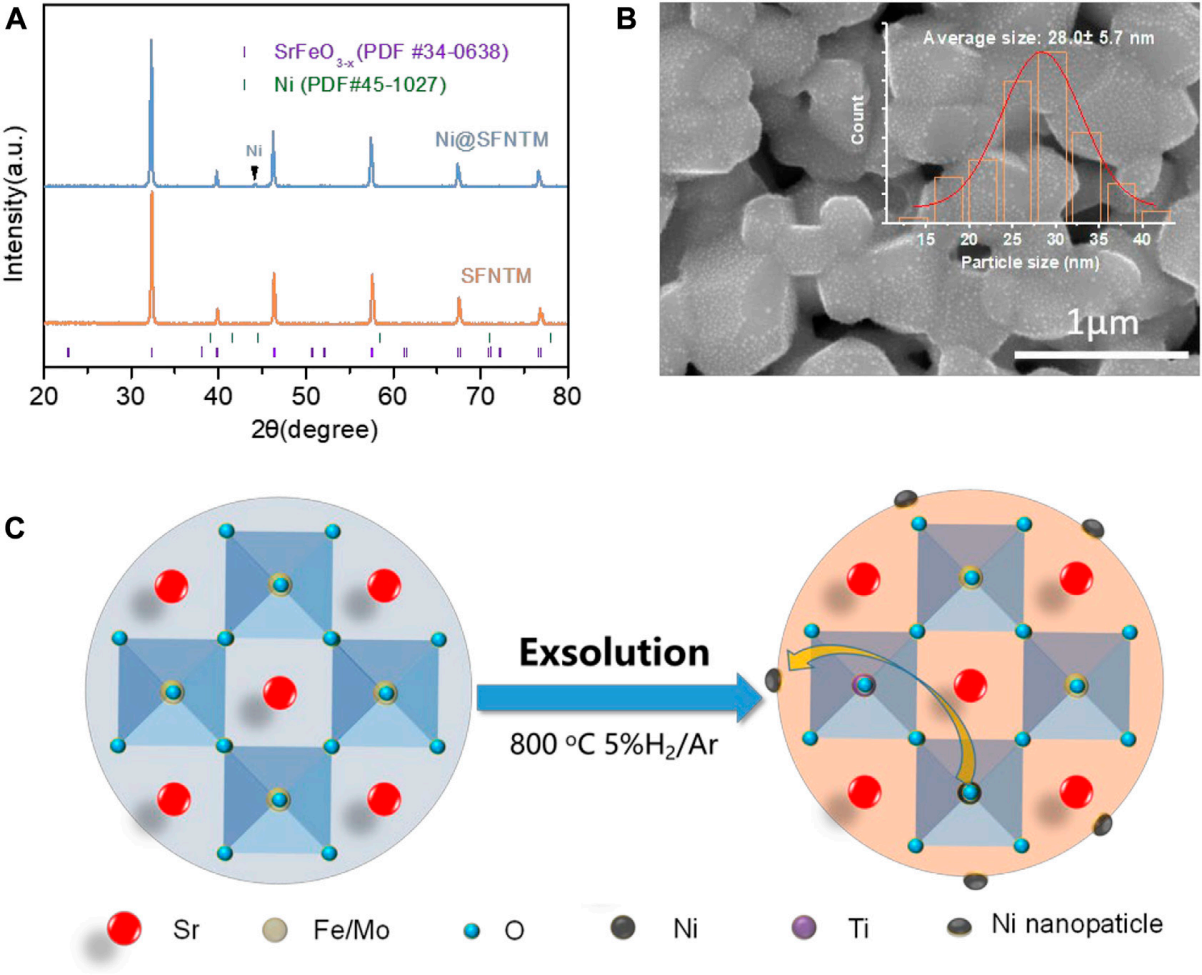
**(A)** XRD patterns of SFTM and Ni@SFNTM powders, **(B)** SEM image of Ni@SFNTM, inset is particle size distribution of Ni nanoparticles, **(C)** Schematic diagram of *in situ* exsolution of SFNTM under reducing conditions.

XRD was used to characterize the crystal structures of the SFNTM and Ni@SFNTM samples at room temperature. Figure 1A shows the XRD pattern of the SFNTM sample after calcination at 1,100^°^C for 5 h and the XRD pattern of the Ni@SFNTM sample after reducing SFNTM in 5% H_2_/Ar at 800^°^C for 5 h. The SFNTM sample presents a pure cubic perovskite structure, which is consistent with that of the PDF card (PDF #34–0638) with no impurity phase; this result confirms that Ti and Ni were successfully co-doped into the perovskite structure. It should be noted that Ni@SFNTM maintains the cubic perovskite structure after the reducing treatment, which is consistent with the Rietveld refinement data (Supplementary Figure S1 and Supplementary Table S1). At the same time, there is an obvious Ni element peak in the XRD pattern of the Ni@SFNTM sample, which is consistent with that of the PDF card (PDF #34–0638), indicating that Ni can be exsolved *in-situ* from the SFNTM matrix under reducing conditions. Furthermore, the (110) peak of Ni@SFNTM shifts to a reduced angle, showing lattice expansion that results from the reduction of the B-site transition metal and the generation of oxygen vacancies. A small peak corresponding to Ni metal is observed for the Ni@SFNTM sample, showing that Ni can be exsolved *in situ* from the SFNTM matrix (Figure 1C). SEM analysis was used to observe the microstructures of the SFNTM and Ni@SFNTM particles. Figure 1B and Supplementary Figure S2 show that the SFNTM particles are interconnected with a smooth surface. After the reduction treatment, Ni@SFNTM presents a glossy surface, while spherical Ni nanoparticles with a mean size of 28 nm are uniformly distributed on the surface of the SFNTM perovskite substrate.

**FIGURE 2:**
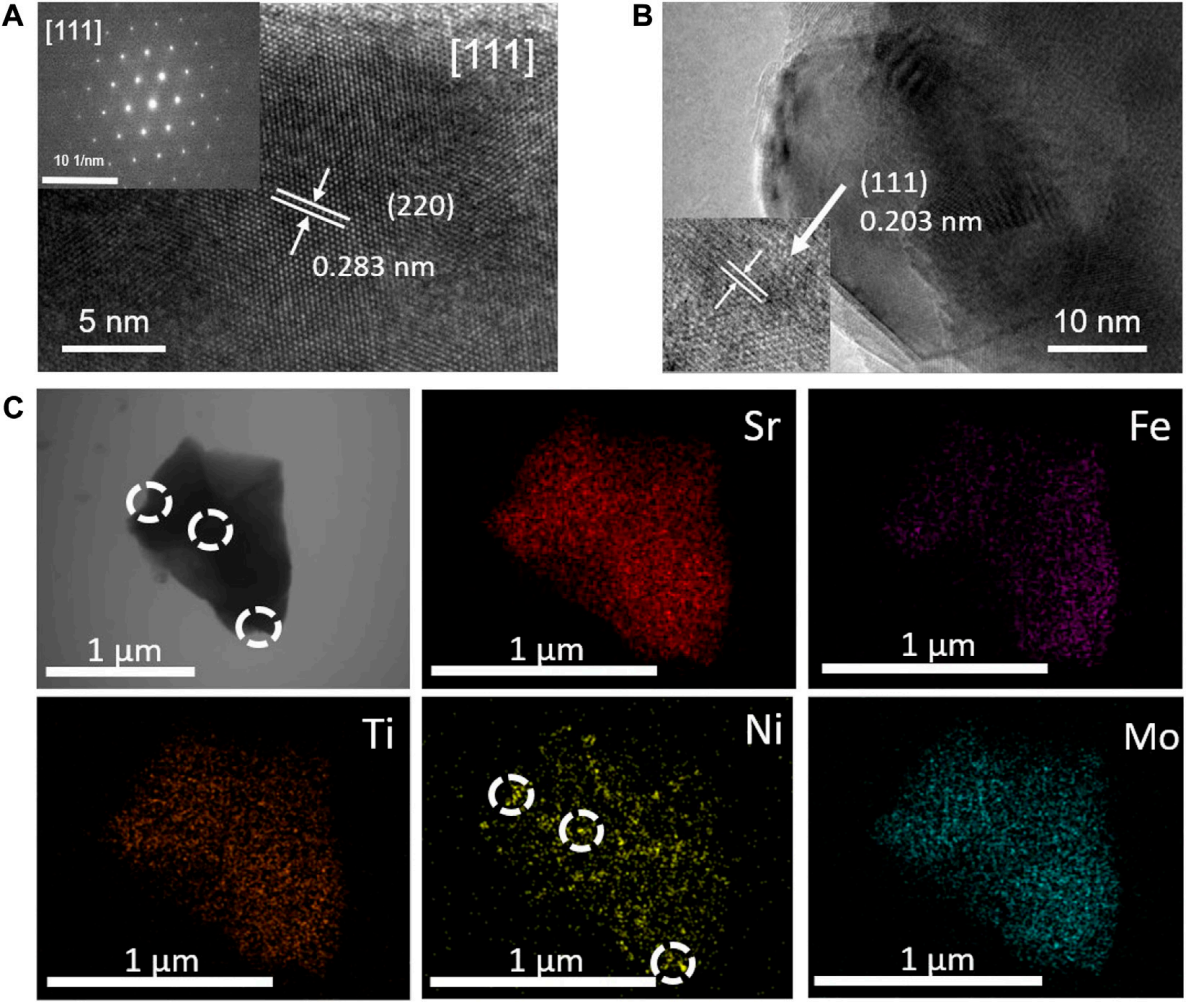
**(A)** HRTEM images and corresponding SEAD image patterns of SFNTM substrate; **(B)** HRTEM images of Ni nanoparticles; **(C)** STEM image and corresponding EDS maps of Ni@SFNTM

To further explore the composition and crystal structure of the synthesized samples, HRTEM characterization was performed on the Ni@SFNTM powder. As shown in Figure 2A, the SAED pattern comprises an image of the particles along the (111) crystal axis, which further confirms that the crystal structure of the SFNTM substrate is a cubic perovskite with a spatial group of Fm-3m. The observed lattice fringe distance of 0.283 nm corresponds to the (220) facet of the cubic SFNTM perovskite, which agrees with the XRD data. Moreover, as shown in Figure 2B, the interplanar spacing (0.203 nm) of the nanoparticles is assigned to the (111) plane of the Ni metal. The spherical Ni nanoparticles are partially anchored in the SFNTM substrate, suggesting extended catalytic active sites and strong bonding between the nanoparticles and the substrate, which greatly enhances the chemical and thermal stability. EDS mapping was performed to further determine the elemental composition of the Ni@SFNTM (Figure 2C). All the elements in the SFNTM substrate are evenly distributed. Exsolved Ni nanoparticles are also found on the surface of the SFNTM substrate particle.

**FIGURE 3:**
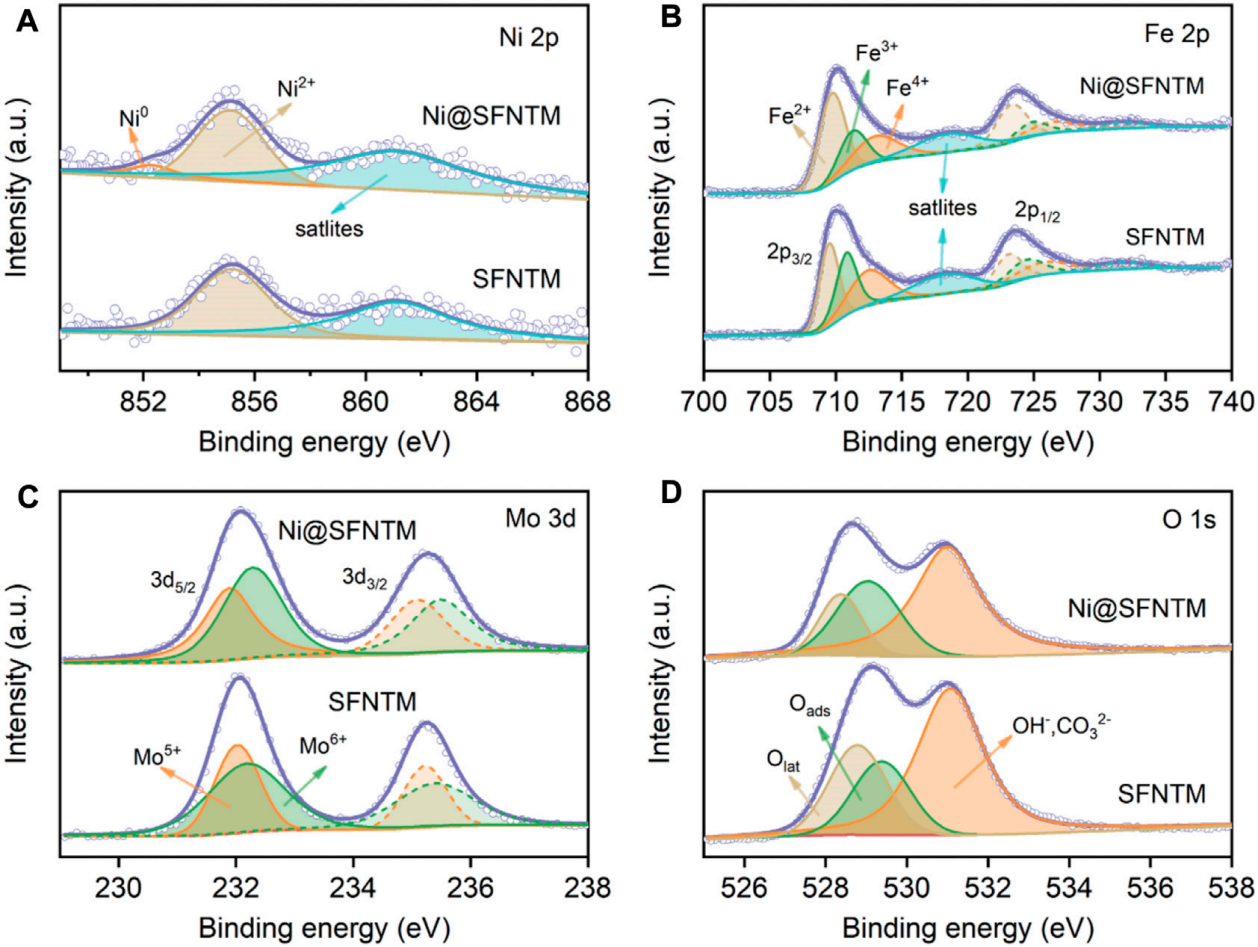
XPS characterization of SFNTM and Ni@SFNTM samples: **(A)** Ni 2p spectra, **(B)** Fe 2p spectra, **(C)** Mo 3 d spectra, **(D)** O 1s spectra.

XPS was performed to determine the valence state changes of the surface elements in the as-prepared and reduced SFNTM samples. Figure 3A and Supplementary Table S2 show the Ni 2p spectra of the samples, before and after the reduction treatment. The figure shows a distinct Ni^0^ characteristic peak at ∼852.2 eV in the Ni@SFNTM sample, further proving that Ni metal particles can be exsolved from the perovskite substrate under a reducing atmosphere. Ni^2+^ peaks are also observed in the spectra, showing that only some Ni metal particles are exsolved from the substrate. Figures 3B,C show the XPS spectra of Fe 2p and Mo 3 d for the Ni@SFNTM and SFNTM samples at room temperature. The peaks at 709.7, 711.3 and 713.0 eV belong to Fe^2+^, Fe^3+^, and Fe^4+^, respectively (Zhang B.-W. et al., 2022). As shown in Supplementary Table S3, both the Fe^3+^ and Fe^4+^ contents decrease and the Fe^2+^ content increases after reduction; the average valence of Fe decreases from 3.00 to 2.79. Moreover, the Mo 3 d spectrum presents a spin-orbit doublet structure, belonging to a mixed state of Mo^5+^ and Mo^6+^. It can also be seen in Supplementary Table S4 that the Mo^6+^/Mo^5+^ ratio decreases after the reduction treatment and the average valence decreases from 5.60 to 5.55, indicating the generation of oxygen vacancies in the SFNTM substrate. The elemental valence states of the Ni, Fe, and Mo transition metals strongly affect the electrocatalytic activity and ionic conductivity of the two samples. The binding energy distributions of the O 1s spectrum are shown in Figure 3D. There are three distinct peaks in the O 1s spectrum corresponding to lattice oxygen (O_lat_), adsorbed oxygen species (O_ads_), and hydroxyl or carbonate (OH^−^/CO_3_^2−^) on the powder surface (Zhou et al., 2018; Liu et al., 2021). From the fitting results (Supplementary Table S5), it can be seen that the ratio of O_ads_/O_lat_ increases from 19.5% to 30.5% after reduction, indicating that the oxygen vacancy concentration and the adsorbed oxygen content on the sample surface are enhanced, promoting the electrochemical reaction process at the electrode.

**FIGURE 4:**
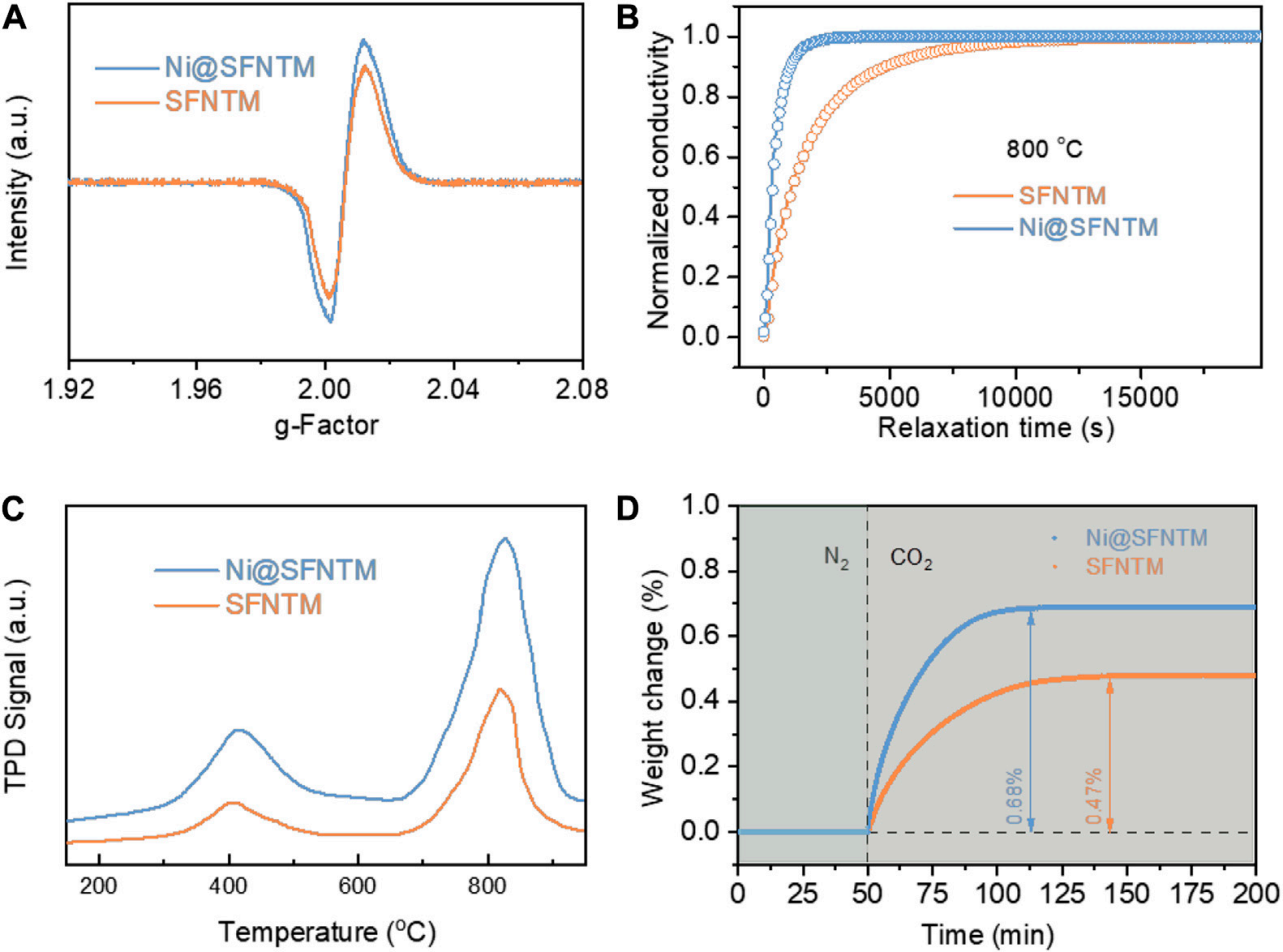
**(A)** EPR measurements and **(B)** ECR curves of SFNTM and Ni@SFNTM samples, **(C)** CO_2_-TPD curves at 800°C, tested after abrupt change of the atmosphere from 2:1 CO-CO_2_ to 1:1 CO-CO_2_, **(D)** Thermogravimetric relaxation curves on changing test gas from N_2_ to CO_2_ at 600°C.

Electron Paramagnetic Resonance (EPR) analysis was used to determine the relative peak intensities of the SFNTM and Ni@SFNTM samples, which correspond to the spin numbers of the lone electrons. Ni@SFNTM shows a higher relative peak intensity than that of SFNTM (Figure 4A), indicating that Ni@SFNTM has a higher concentration of oxygen vacancies; this result is consistent with the XPS results of the O 1s spectra (Figure 3D) (Xi et al., 2021) Moreover, the surface oxygen exchange and bulk diffusion properties of the two samples were evaluated using the ECR method, which can imply the kinetics of the surface CO_2_-RR and bulk diffusion in a CO_2_-rich atmosphere (Zhu et al., 2016; Li et al., 2019). Figure 4B shows that the oxygen exchange rate can reach equilibrium state with an increase in the CO_2_ concentration. The shorter the relaxation time, the faster the oxygen exchange rate. The Ni@SFNTM sample presents a shorter relaxation time than the SFNTM sample at 800°C, implying that the Ni@SFNTM sample has a faster oxygen ion exchange rate due to the increased oxygen vacancies after reduction and can speed up the oxygen transfer process. Additionally, the calculated surface exchange coefficients (*K*_chem_) of SFNTM and Ni@SFNTM are 2.42 × 10^–5^ cm s^−1^ and 8.67 × 10^–5^ cm s^−1^, respectively. The corresponding chemical bulk diffusion coefficients (*D*_chem_) are 2.58 × 10^–6^ cm^2^ s^−1^ for SFNTM and 7.85 × 10^–6^ cm^2^ s^−1^ for Ni@SFNTM. In SOECs, the CO_2_ chemisorption process on the surface of the cathode is a rate-limiting step for CO_2_-RR, where the adsorption capacity of CO_2_ is crucial for the cathode materials of SOEC (Lv et al., 2021). The CO_2_ adsorption properties of Ni@SFNTM and SFNTM were determined using CO_2_-TPD measurements (Figure 4C). Two desorption peaks at ∼400 and ∼800^°^C can be clearly observed in the desorption process of CO_2_ corresponding to the physical desorption and the chemical desorption, respectively. At low temperatures, both samples exhibit similar physical desorption processes (Ye et al., 2017). However, both the chemical desorption peak and the corresponding peak area of Ni@SFNTM are higher than those of SFNTM at high temperatures, indicating an enhanced strong binding force for CO_2_ adsorption caused by the increased oxygen vacancy and exsolved Ni nano particles. Furthermore, thermogravimetric relaxation was used to investigate the weight changes of the Ni@SFNTM and SFNTM samples by rapidly switching the N_2_ atmosphere to a CO_2_ atmosphere at 600^°^C. Figure 4D shows that the weights of the two samples rapidly increase owing to the adsorption of CO_2_ and reach equilibrium when the adsorption of CO_2_ reached saturation. The Ni@SFNTM sample has a higher weight increase (0.68 %) and shorter adsorption time, indicating that Ni@SFNTM has more oxygen vacancies and stronger CO_2_ chemisorption. This result is in good agreement with the XPS and CO_2_-TPD results.

**FIGURE 5:**
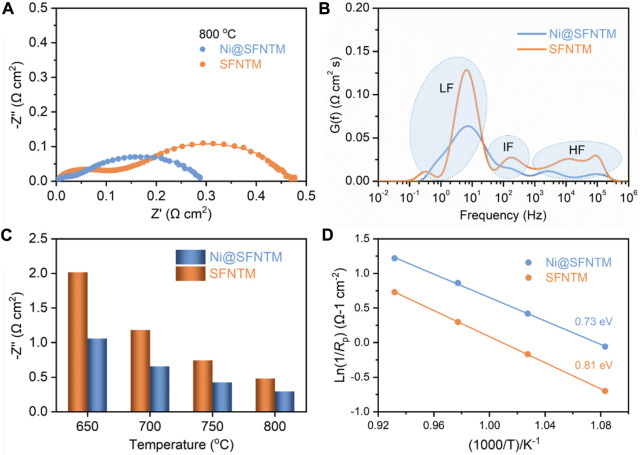
**(A)** EIS patterns of SFNTM and Ni@SFNTM powders at 800°C, and **(B)** corresponding distribution of the relaxation time (DRT) curves, **(C)** Impedance values of two samples from 650 to 800°C, **(D)** CO_2_-RR activation energy at 650–800°C.

In order to study the electrochemical performance and analyze the CO_2_ electrolysis reaction of the two cathode materials, EIS was performed on the LSGM electrolyte-supported single cells with the configuration of SFNTM/Ni@SFTNM|SDC|LSGM|LSCF under a pure CO_2_ atmosphere at an OCV. Figure 5A shows the polarization resistance (*R*_p_) of a single cell with Ni@SFNTM and SFNTM as cathodes at 800^°^C. The *R*_p_ value of Ni@SFNTM is 0.29 Ω cm^−2^, which is much lower than that of SFNTM (0.48 Ω cm^−2^). The EIS data obtained at applied potential of 1.4 V and 800°C were analyzed, as shown in Supplementary Figure S3. Clearly, the SOEC with the Ni@SFNTM cathode significantly reduces the *R*p at 1.4 V, which is consistent with the OCV. Owing to the high catalytic activity of Ni metal and enhanced oxygen vacancies, the reduced *R*_p_ improves the CO_2_-RR activity of Ni@SFNTM. Figure 5B shows the distribution of the relaxation time (DRT) technology used to explore the separation of the pivotal electrode reaction processes and analyze the deconvolution of the EIS data to further understand the CO_2_-RR electrocatalysis process (Zhang et al., 2015; Zhang et al., 2016). There are three areas in the DRT curves of the Ni@SFNTM and SFNTM samples. The peaks in the high-frequency area (HF) are primarily associated with the oxygen evolution reaction in the anode and the transportation of O^2-^ through the interface of the electrodes and electrolyte (Zhou et al., 2018; Jiang et al., 2019). The low-frequency (LF) area is likely attributed to gas adsorption and the dissociation processes on the cathode surface (Li et al., 2017b). Additionally, the intermediate frequency (IF) is between HF and LF, and represents reactant diffusion and surface processes (Chen et al., 2018; Tian et al., 2020). Both IF and LF exhibit kinetic processes on the electrode surface. It is worth noting that the IF and LF of the Ni@SFNTM cathode significantly decreases, demonstrating that the CO_2_ adsorption and electrolysis reaction processes improved. The positions of the characteristic peaks related to IF and LF shift to higher frequencies, suggesting that the electrochemical reaction kinetics should be accelerated. In contrast, after the reduction treatment, the exsolved Ni metal and the increased oxygen vacancies supply more active reaction sites for CO_2_ adsorption and activation, while the formed metal-oxide heterostructure with strong interaction enhances the surface exchange reactions (Lee et al., 2021; Zhang L. H. et al., 2022). This occurrence indicates that the CO_2_-RR performance of the Ni@SFNTM cathode is enhanced. These results are in agreement with the XPS, TDP, and ECR results. Moreover, the *R*p values of the two cathodes at different operating temperatures are investigated as shown in Figure 5C. The polarization resistances of both cathodes decrease with an increase in temperature, and the *R*p values of the Ni@SFNTM cathode are lower than those of the SFNTM cathode at a certain temperature. Furthermore, the lower activation energy of the Ni@SFNTM cathode (Figure 5D) also confirms the improved CO_2_-RR kinetics and enhanced O^2-^ transportation, primarily resulting from the heterostructure and extended reactive area (Lv et al., 2022).

**FIGURE 6:**
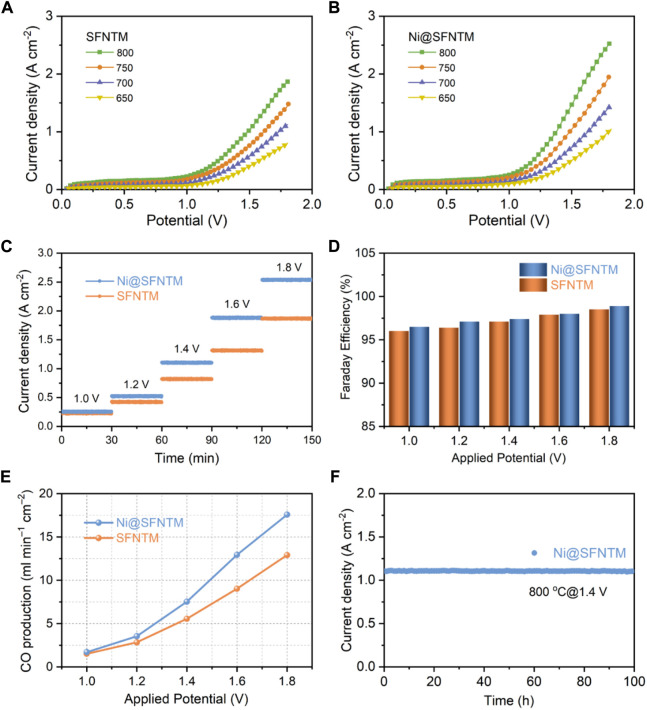
Electrochemical performance of **(A)** SFNTM and **(B)** Ni@SFNTM samples under pure CO_2_ atmosphere, **(C)** Short-term stability under different applied potentials at 800°C, **(D)** Comparison of Faradaic efficiency under different application potentials, and **(E)** corresponding CO production rate, **(F)** Long-term stability of single cell with Ni@SFNTM cathode under pure CO_2_ atmosphere at 1.4 V and 800^°^C.

Consequently, the current-voltage (*I*-*V*) curves of the single cells with SFNTM and Ni@SFNTM cathodes were measured at 650–800°C respectively, (Figures 6A,B). The CO_2_ electrolysis current density of the Ni@SFNTM cathode is 2.54 A cm^−2^ at 1.8 V and 800°C, outperforming the SFNTM cathode (1.86 A cm^−2^). Because of the anodes and electrolytes of the single cells are similar, the improved CO_2_-RR performance can be mainly ascribed to the excellent electrocatalytic activity of the Ni@SFNTM cathode. Moreover, the current densities of Ni@SFNTM are also comparable to those of other perovskite oxide cathodes (Supplementary Table S6). The short-term stability measurements of CO_2_-RR at various voltages are shown in Figure 6C; the Ni@SFNTM-based single cell exhibits an excellent CO_2_-RR performance at different electrolysis potentials. For instance, the current densities of the single cells with Ni@SFNTM cathode are 1.88 and 2.54 A cm^−2^ at 1.6 and 1.8 V, respectively, which is approximately 1.43 and 1.36 times than that of the single cell with the SFNTM cathode (1.31 and 1.87 A cm^−2^). Furthermore, gas chromatographic analysis was used to obtain the CO production rate and corresponding Faradaic efficiency by evaluating the exhaust gas collected at different applied potentials during short-time stability measurements. As shown in Figures 6D,E, both the CO production rate and Faradaic efficiency of the two cells increase with increasing applied potential; the Faradaic efficiency of both cells is close to 100%, demonstrating that CO is the primary product and there is no coke deposition during CO_2_ electrolysis. The CO generation rate of the Ni@SFNTM-based single cell is 12.5 ml min^−1^ cm^−2^ at 1.6 V, approximately 1.56 times that of the SFNTM-based single cell (8 ml min^−1^ cm^−2^). This further demonstrates that the formed metal-oxide heterostructure significantly enhances the CO_2_-RR performance. Additionally, the single cell with the Ni@SFNTM cathode exhibits excellent long-term operational stability. Figure 6F shows that a stable current density is maintained for 100 h under an applied potential of 1.4 V at 800^°^C in a pure CO_2_ atmosphere. These results indicate that Ni@SFNTM with a Ni metal and SFNTM oxide heterostructure is a potential SOEC cathode for highly efficient CO_2_ electrolysis.

## Conclusion

In this study, Ti and Ni co-doped SFM perovskite oxides were prepared and used as cathodes for the direct electrolysis of CO_2_ in SOECs. A metal-oxide heterostructure was obtained after a reducing treatment in 5% H_2_/Ar at 800°C, providing more reactive sites for CO_2_-RR, enhancing the chemisorption and activation capacity of CO_2_, and significantly improving electrolysis performance. Moreover, the single cell with Ni@SFNTM cathode presented a large CO_2_ electrolysis current density of 2.54 A cm^−2^ at 1.8 V and 800°C, exceeding that of the cell comprising the SFNTM cathode (1.87 A cm^−2^ at 1.8 V); it also exhibits excellent long-term stability. This improved performance is primarily attributed to the metal-oxide heterostructure and abundant oxygen vacancies created by the reduction treatment and the strong interaction between the metal and oxide. These results indicate that Ni@SFNTM with the Ni metal and SFNTM oxide heterostructure is a potential SOEC cathode for the efficient electrolysis of CO_2_, and this method provides a common strategy for designing high performance electrode materials for SOECs.

## Data Availability

The original contributions presented in the study are included in the article/Supplementary Material, further inquiries can be directed to the corresponding authors.
