# Supplementary material for: Ti/Ni co-doped perovskite cathode with excellent catalytic activity and CO2 chemisorption ability via nanocatalysts exsolution for solid oxide electrolysis cell
Source: Front Chem. 2022 Oct 10;10:1027713. doi: 10.3389/fchem.2022.1027713 (PMC9589057; doi:10.3389/fchem.2022.1027713)
Supplement: Supplementary file 1 [file DataSheet1.docx]

Supplementary Material

# Supplementary Figures and Tables

## Supplementary Figures


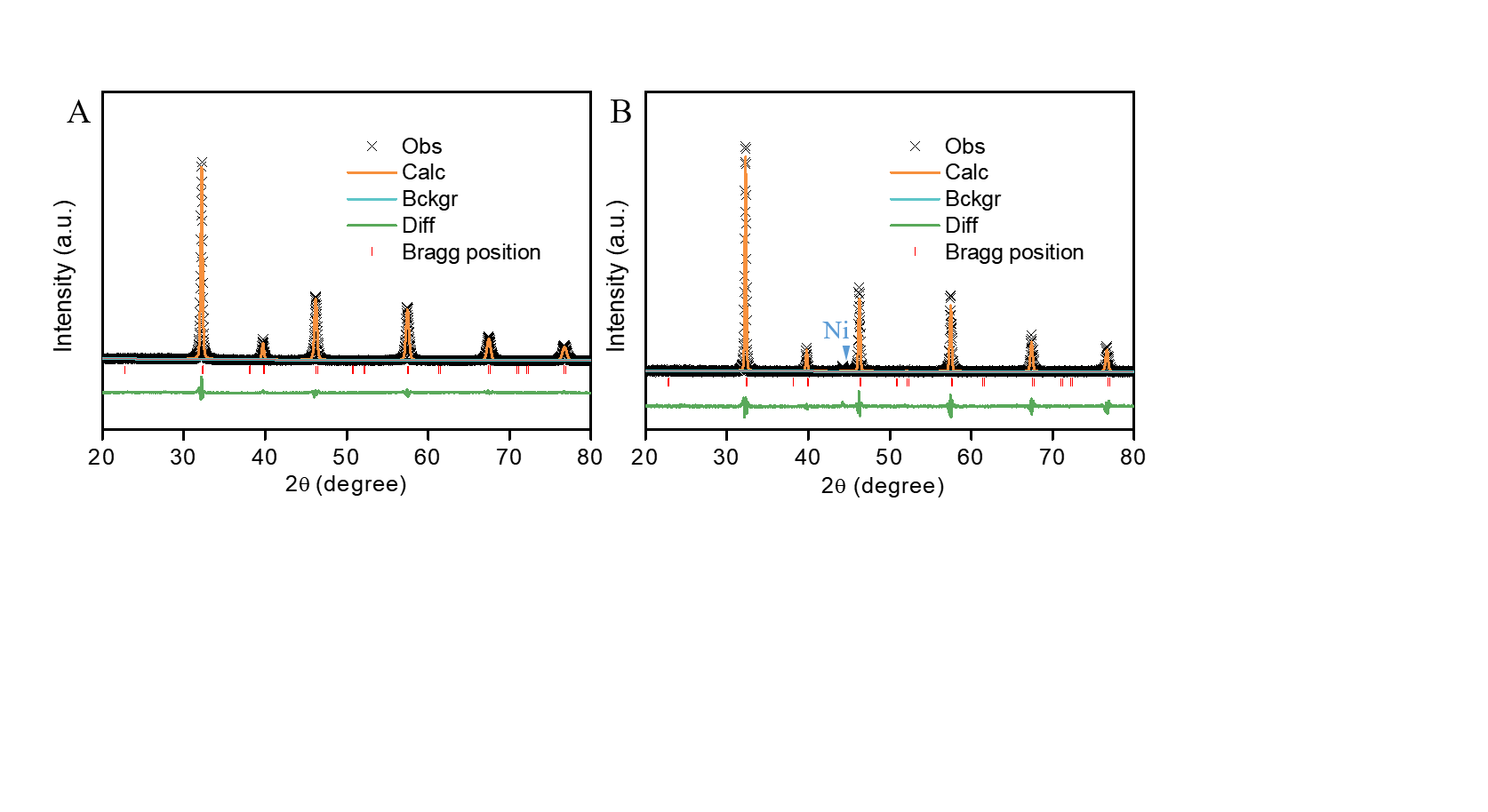


**Figure S1.** Rietveld refined XRD patterns of the (A) SFNTM sample and (B) Ni@SFNTM sample


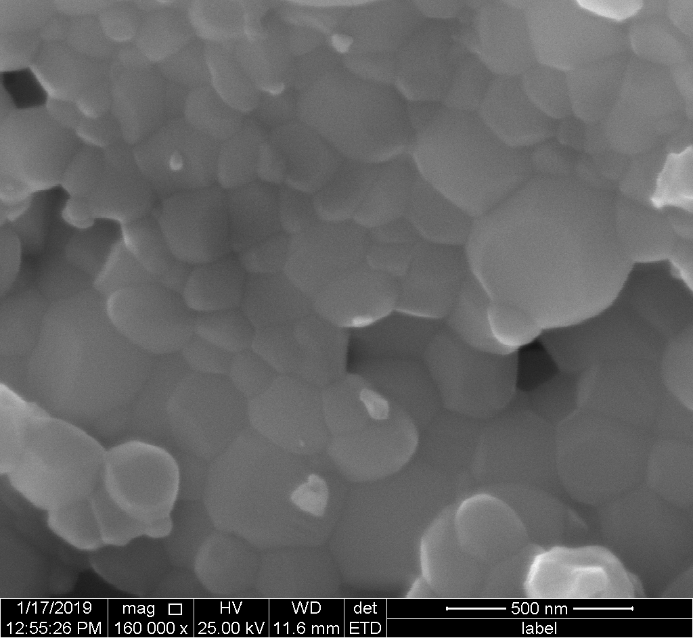


**Figure S2.** SEM image of SFNTM sample

**
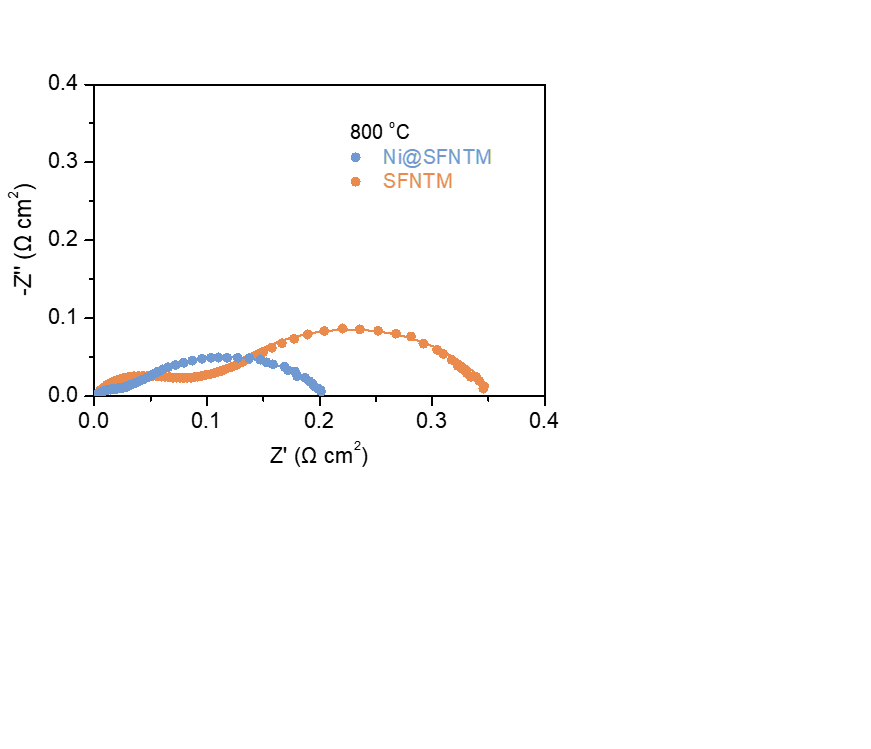
**

**Figure S3.** EIS patterns of SFNTM and Ni@SFNTM powders at 1.4 V and 800 °C

## Supplementary Tables

**Table S1** XRD refined data of SFNTM and Ni@SFNTM samples

| Samples | a(Å) | b(Å) | c(Å) | V(Å) | Rp | Rwp | χ2 |
| --- | --- | --- | --- | --- | --- | --- | --- |
| SFNTM | 7.816 | 7.816 | 7.816 | 477.5 | 0.0672 | 0.0813 | 1.799 |
| Ni@SFNTM | 7.898 | 7.898 | 7.898 | 492.76 | 0.0014 | 0.003 | 0.1977 |

**Table S2** The valence state distribution of Ni in SFNTM and Ni@SFNTM samples

|  | SFNTM (%) | Ni@SFNTM (%) |
| --- | --- | --- |
| Ni^0^ | 0 | 15.5 |
| Ni^2+^ | 100 | 84.5 |
| Average valence | 2 | 1.69 |

**Table S3** The valence state distribution of Fe in SFNTM and Ni@SFNTM samples

|  | SFNTM (%) | Ni@SFNTM (%) |
| --- | --- | --- |
| Fe^2+^ | 34.0 | 48.9 |
| Fe^3+^ | 32.2 | 23.2 |
| Fe^4+^ | 33.8 | 27.9 |
| Average valence | 3.00 | 2.79 |

**Table S4** The valence state distribution of Mo in SFNTM and Ni@SFNTM samples

|  | SFNTM | Ni@SFNTM |
| --- | --- | --- |
| Mo^5+^ | 39.7% | 45.2% |
| Mo^6+^ | 60.3% | 54.8% |
| Mo^6+^/Mo^5+^ | 1.52 | 1.21 |
| Average valence | 5.60 | 5.55 |

**Table S5** The valence state distribution of O in in SFNTM and Ni@SFNTM samples

|  | SFNTM | Ni@SFNTM |
| --- | --- | --- |
| O_lat_ (O^2-^) | 22.7% | 14.9% |
| O_ads_(O^−^/O_2_^2−^) | 18.5% | 30.5% |
| OH^−^/CO_3_^2−^ | 58.8% | 54.6% |
| O_ads_/O_lat_ | 0.81 | 2.05 |

**Table S6.** The comparison of different cells for CO_2_ electrolysis under similar operating conditions at 1.6V and 800°C

| Cathodes | Electrolytes | Anodes | I (A cm^−2^) | Refs |
| --- | --- | --- | --- | --- |
| Sr_2_Fe_1.5_Mo_0.5_O_6-δ_-GDC | YSZ | LSM-YSZ | 0.45 | (Song et al., 2019) |
| Sr_2_Fe_1.4_Mn_0.1_Mo_0.5_O_6−δ_/SDC | LSGM | LSCF/SDC | ∼1.42 | (Jiang et al., 2019) |
| NiFe_3_@Sr_2_Fe_1.33_Mo_0.45_Ni_0.2_O_6−δ_/GDC | LSGM | LSCF/GDC | 0.93 | (Lv, Lin, et al., 2019) |
| Sr_2_Fe_1.5_Mo_0.5_O_6−δ_F_0.1_ | LSGM | LSCF/SDC | 1.6 | (Li et al., 2019) |
| CoFe@Sr_2_Fe_1.35_Mo_0.45_Co_0.2_O_6−δ_/GDC | LSGM | BSCF/GDC | 1.05 | (Lv et al., 2020) |
| Sr_2_FeMoO_6−δ_ | LSGM | LSCF/SDC | ∼0.70 | (Xi, Liu, Fan, et al., 2021) |
| Sr_2_Fe_1.3_Zr_0.2_Mo_0.5_O_6-δ_ | LSGM | LSCF | 1.09 | (Zhang et al., 2022) |
| Sr_2_Fe_1.5_Mo_0.5_O_6-δ_-CaCO_3_ | LSGM | LSCF | 1.09 | (Zhang et al., 2022) |
| SFNTM | LSGM | LSCF/SDC | 1.31 | This work |
| Ni@SFNTM | LSGM | LSCF/SDC | 1.88 | This work |

**Reference**

Song, Y. F., X. M. Zhang, Y. J. Zhou, H. F. Lv, Q. X. Liu, W. C. Feng, G. X. Wang, and X. H. Bao. (2019). Improving the performance of solid oxide electrolysis cell with gold nanoparticles-modified LSM-YSZ anode, *Journal of Energy Chemistry*, 35: 181-87. doi.org/10.1016/j.jechem.2019.03.013.

Jiang, Yunan, Yi Yang, Changrong Xia, and Henny J. M. Bouwmeester. (2019). Sr_2_Fe_1.4_Mn_0.1_Mo_0.5_O_6−δ_ perovskite cathode for highly efficient CO_2_ electrolysis, *Journal of Materials Chemistry A*, 7: 22939-49. doi.org/10.1039/c9ta07689a.

Lv, H. F., L. Lin, X. M. Zhang, D. F. Gao, Y. F. Song, Y. J. Zhou, Q. X. Liu, G. X. Wang, and X. H. Bao. (2019). In situ exsolved FeNi_3_ nanoparticles on nickel doped Sr_2_Fe_1.5_Mo_0.5_O_6-δ_ perovskite for efficient electrochemical CO_2_ reduction reaction, *Journal of Materials Chemistry A*, 7: 11967-75. doi.org/10.1039/c9ta03065d.

Li, Yihang, Yong Li, Yanhong Wan, Yun Xie, Junfa Zhu, Haibin Pan, Xusheng Zheng, and Changrong Xia. (2019). Perovskite Oxyfluoride Electrode Enabling Direct Electrolyzing Carbon Dioxide with Excellent Electrochemical Performances, *Advanced Energy Materials*, 9: 1803156. doi.org/https://doi.org/10.1002/aenm.201803156.

Lv, Houfu, Le Lin, Xiaomin Zhang, Yuefeng Song, Hiroaki Matsumoto, Chaobin Zeng, Na Ta, Wei Liu, Dunfeng Gao, Guoxiong Wang, and Xinhe Bao. (2020). In Situ Investigation of Reversible Exsolution/Dissolution of CoFe Alloy Nanoparticles in a Co-Doped Sr_2_Fe_1.5_Mo_0.5_O_6−δ_ Cathode for CO_2_ Electrolysis, *Advanced Materials*, 32: 1906193. doi.org/https://doi.org/10.1002/adma.201906193.

Xi, Xiuan, Jianwen Liu, Yun Fan, Lijuan Wang, Jun Li, Mingming Li, Jing-Li Luo, and Xian-Zhu Fu. (2021). Reducing d-p band coupling to enhance CO_2_ electrocatalytic activity by Mg-doping in Sr_2_FeMoO_6-δ_ double perovskite for high performance solid oxide electrolysis cells, *Nano Energy*, 82: 105707. doi.org/https://doi.org/10.1016/j.nanoen.2020.105707.

Zhang, L. H., C. M. Xu, W. Sun, R. Z. Ren, X. X. Yang, Y. Z. Luo, J. S. Qiao, Z. H. Wang, S. Y. Zhen, and K. N. Sun. (2022). Constructing perovskite/alkaline-earth metal composite heterostructure by infiltration to revitalize CO_2_ electrolysis, *Separation and Purification Technology*, 298: 9. doi.org/10.1016/j.seppur.2022.121475.

Zhang, L. H., W. Sun, C. M. Xu, R. Z. Ren, X. X. Yang, J. S. Qiao, Z. H. Wang, S. Y. Zhen, and K. N. Sun. (2022). Two-fold improvement in chemical adsorption ability to achieve effective carbon dioxide electrolysis, *Applied Catalysis B: Environmental*, 317: 121754. doi.org/10.1016/j.apcatb.2022.121754.
